# Freeze-Dried Platelet-Rich Plasma Accelerates Bone Union with Adequate Rigidity in Posterolateral Lumbar Fusion Surgery Model in Rats

**DOI:** 10.1038/srep36715

**Published:** 2016-11-11

**Authors:** Yasuhiro Shiga, Sumihisa Orita, Go Kubota, Hiroto Kamoda, Masaomi Yamashita, Yusuke Matsuura, Kazuyo Yamauchi, Yawara Eguchi, Miyako Suzuki, Kazuhide Inage, Takeshi Sainoh, Jun Sato, Kazuki Fujimoto, Koki Abe, Hirohito Kanamoto, Masahiro Inoue, Hideyuki Kinoshita, Yasuchika Aoki, Tomoaki Toyone, Takeo Furuya, Masao Koda, Kazuhisa Takahashi, Seiji Ohtori

**Affiliations:** 1 Department of Orthopaedic Surgery, Graduate School of Medicine, Chiba University, Chiba, Japan; 2 Department of Orthopaedic Surgery, Eastern Chiba Medical Center, Chiba, Japan; 3 Department of Orthopaedic Surgery, Showa University, Tokyo, Japan

## Abstract

Fresh platelet-rich plasma (PRP) accelerates bone union in rat model. However, fresh PRP has a short half-life. We suggested freeze-dried PRP (FD-PRP) prepared in advance and investigated its efficacy *in vivo*. Spinal posterolateral fusion was performed on 8-week-old male Sprague-Dawley rats divided into six groups based on the graft materials (n = 10 per group): sham control, artificial bone (A hydroxyapatite–collagen composite) –alone, autologous bone, artificial bone + fresh-PRP, artificial bone + FD-PRP preserved 8 weeks, and artificial bone + human recombinant bone morphogenetic protein 2 (BMP) as a positive control. At 4 and 8 weeks after the surgery, we investigated their bone union–related characteristics including amount of bone formation, histological characteristics of trabecular bone at remodeling site, and biomechanical strength on 3-point bending. Comparable radiological bone union was confirmed at 4 weeks after surgery in 80% of the FD-PRP groups, which was earlier than in other groups (p < 0.05). Histologically, the trabecular bone had thinner and more branches in the FD-PRP. Moreover, the biomechanical strength was comparable to that of autologous bone. FD-PRP accelerated bone union at a rate comparable to that of fresh PRP and BMP by remodeling the bone with thinner, more tangled, and rigid trabecular bone.

*Statement of clinical significance:* FD-PRP provides bone union ability comparable to that of fresh PRP and BMP, and can be prepared in advance safely and cheaply.

In orthopedic surgery for conditions such as fracture and unstable spinal pathology, stabilization of the unstable segments through rigid bone union is very important and is one of the major objectives of arthrodesis surgery^1^,^2^,^3^.

Generally, postoperative bone union takes at least a few weeks. However, occasional delayed and inadequate bone union causes pseudoarthrosis, which can lead to chronic instability, and pain as well as poor activities of daily living and quality of life^4^. Thus, accelerated postoperative bone union is urgently needed. Among the materials used for bone union, platelet-rich plasma (PRP) has the potensial, with its known acceleration for tissue healing in the field of plastic surgery and dentistry^5^,^6^,^7^. However, only a few studies have reported on its efficacy on bone union in orthopedic surgery^8^,^9^.

In our previous studies, PRP administration in rat spinal fusion models significantly led to faster bone union without any complication^10^,^11^. This superior ability to accelerate bone fusion can be applied to other orthopedic arthrodesis surgeries^12^,^13^,^14^,^15^. However, one of the possible barriers to the clinical application of fresh PRP is its short half-life of merely several days^16^. To overcome such issues, freeze-dried PRP (FD-PRP) has been suggested^17^.

FD-PRP is reported to retain growth factors after preparation^18^, and clinical trial combined with bone allograft has been performed to report its usefulness for periodontal endosseous defects^19^. However, the clinical application of FD-PRP in spinal arthrodesis surgery has not yet been investigated. Furthermore, there are no reports of clinical applications of FD-PRP mixed with artificial bone materials such as hydroxyapatite and collagen, which are generally used as carriers and scaffolds in arthrodesis surgeries for more effective bone union.

In the current study, we examined the efficacy and properties of FD-PRP in bone union with artificial bone materials using lumbar arthrodesis models in rats.

## Materials and Methods

### Experimental Animals

Male Sprague-Dawley rats weighing 250 to 300 g were used in this study. All protocols for animal procedures were approved by the ethics committees of Chiba University and followed the National Institutes of Health Guidelines for the Care and Use of Laboratory Animals (1996 revision). The protocols for experimental animals were based on our previous study^10^.

### FD-PRP Preparation

In the current study, we used allograft blood for the PRP instead of autograft. Ten out of a total of 60 rats were used as blood donors for PRP preparation. After they were anesthetized, approximately 15 mL of fresh blood was transcardially obtained using a syringe containing 2 mL of acid–citrate–dextrose solution A (Terumo, Tokyo, Japan) to prevent coagulation. The whole blood was centrifuged (KN70; Kubota, Tokyo, Japan) at 1500 rpm for 10 minutes. Subsequently, the plasma fraction was separated from the red blood cells and further centrifuged at 3000 rpm for 10 minutes to pellet the platelets as previously described^6^. The pelleted platelets were collected and separated from the supernatant platelet-poor plasma (PPP). PRP was generated by mixing the platelets with 2.5 mL of PPP.

**Figure 1:**
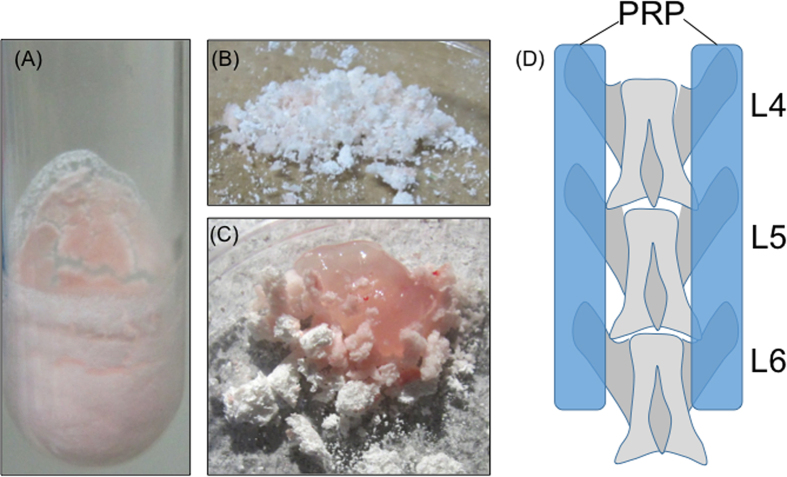
(**A**) FD-PRP preparation. FD-PRP appears as powder, which can be dissolved in distilled water with the same concentration of fresh PRP. (**B**) The artificial bone is crushed into powder. (**C**) FD-PRP is mixed with the powdered artificial bone followed by activation using thrombin and calcium chloride before use. (**D**) Schema of the spine (transplantation site). The graft material was implanted over the transverse processes of L4–L6.

Each of the PRP aliquots was weighed prior to freeze-drying. The test tubes were then rotated in an ethanol bath at −60 °C for membranous freezing (preliminary freezing) and then immediately frozen at −30 °C for 4 h. The tubes were then attached to a vacuum freeze-dryer to complete the process and stored for 8 weeks at 4 °C. The FD-PRP samples were resuspended in distilled water prior to assessment. To avoid any changes in component concentrations, the weight after dissolving was matched to the weight before freeze-drying (Fig. 1A).

PRP requires activation before application by adding calcium chloride solution (1 mEq/mL; Otsuka Pharmaceutical, Tokyo, Japan) and thrombin solution (Mochida Pharmaceutical, Tokyo, Japan) to each FD-PRP sample. Each amount is one-tenth of the amount of FD-PRP. The platelet counts in the whole blood, fresh PRP, and FD-PRP were determined with a hematology analyzer.

### Lumbar Posterolateral Fusion Models

Spinal surgery was performed in 50 eight-week-old male Sprague-Dawley rats. Ten rats were allocated to the sham group, whose L4–6 transverse processes only exposed but received no treatment and 50 rats underwent bilateral posterolateral fusion (PLF). We divided the 50 rats into groups of 10 based on the graft material used: artificial bone group (artificial bone–alone), autologous bone group, artificial bone treated with fresh-PRP (fresh-PRP) group, and artificial bone with FD-PRP (FD-PRP) group. Positive control was also prepared using human recombinant bone morphogenetic protein 2 and artificial bone (BMP group). A hydroxyapatite –collagen composite, Refit^®^ (Hoya Corporation, Tokyo, Japan), was used as the artificial bone graft substitute. The FD-PRP group used a mixture of 0.5 mL Refit and 0.5 mL gel-activated FD-PRP (Fig. 1B,C). In the BMP group, 0.5 mL Refit and 5 μg of BMP (Sigma-Aldrich Corporation, St. Louis, MO, USA) were mixed and transplanted^20^,^21^. In the autologous bone group, the rats were implanted with ground spinous processes of T10–L2. All of the various graft types were standardized to equal volumes. Corticotomy was not performed in any group.

The bilateral posterolateral lumbar spine in each rat was exposed through a midline skin incision followed by two paramedian fascial incisions using blunt dissection to expose the bilateral lamina and the transverse processes of L4–L6 (Fig. 1D). These graft materials had equal volume measured by 500 mm^3^ of Cryomold (Sakura Finetek Japan Corporation, Tokyo, Japan).

### Determination of Growth Factor Concentrations

To confirm the activity of PRP, concentrations of platelet-derived growth factor BB (PDGF-BB) and transforming growth factor β1 (TGF-β1) in blood, activated PRP, and activated FD-PRP were measured with enzyme-linked immunosorbent assay (ELISA) technique using a Quantikine ELISA kit (R&D Systems, Minneapolis, MN, USA). The immunoassays were performed following the manufacturer’s instructions.

### Radiographic Examination (Evaluation of Bone Union)

To evaluate bone union, rats were anesthetized with sodium pentobarbital (40 mg/kg, i.p.). Then lumbar radiographs were obtained in the anteroposterior aspect at 4 and 8 weeks after the surgery (Bruker Corp., Billerica, MA, USA). Bone union was considered to be achieved when the transverse processes of L4–L6 were bridged. Bone union was determined by three independent observers who were blinded to the experiment, and union was accepted if all three observers agreed.

### Histological Examination

After radiographic examination at 8 weeks, we randomly selected half of the rats in each group (n = 5 per group), which were perfused transcardially with 300 mL of 4% paraformaldehyde in phosphate buffer (0.1 M, pH 7.4). The L4–L6 lumbar vertebrae were harvested and immersed in 4% paraformaldehyde phosphate buffer solution. The coronal sections including the transverse processes of each level were histologically evaluated for the extent bone formation and intrabone structure (trabecular bone).

### Amount of Bone Formation

**Figure 2:**
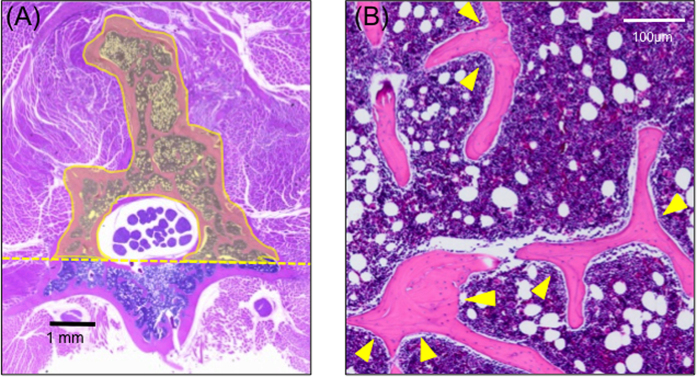
Histological image of the lumbar spine (hematoxylin and eosin stain). (**A**) Dashed line: horizontal line at the lower edge of the dural sac. We have measured the entire remodeling part above the dotted line, including the upper part of the vertebral arch and the spinous process. (**B**) Arrowheads indicate the trabecular bone branches.

Histological area measurement was done using ImageJ software (NIH). To eliminate the error of the magnitude of the vertebral body of each specimen, the amount of bone formation was measured at the horizontal line at the lower (ventral) and upper (dorsal) edges of the spinal column. We have measured the entire remodeling part, including the upper part of the vertebral arch and the spinous process above the line mentioned above (Fig. 2A).

### Trabecular Bone Evaluation

#### Total area ratio

Two remodeling spots on the left and right sides sandwiching the spinous process of the trabecular bone were selected randomly for evaluation. The percentage of trabecular bone area in the whole field of view was measured using Image-J software, and the average values for the left and right sides were obtained to calculate the ratio to the whole area.

#### Number of trabecular branches

The total number of trabecular branches was counted and statistically evaluated (Fig. 2B).

#### Width of each trabecular branches

We randomly chose five trabecular bones with linear portions, and measured the average width of each trabecular bone at three points; both ends and the middle point within the part of interest, and their mean values were calculated and statistically compared among the groups.

#### Mechanical strength examination (three-point bending test)

**Figure 3:**
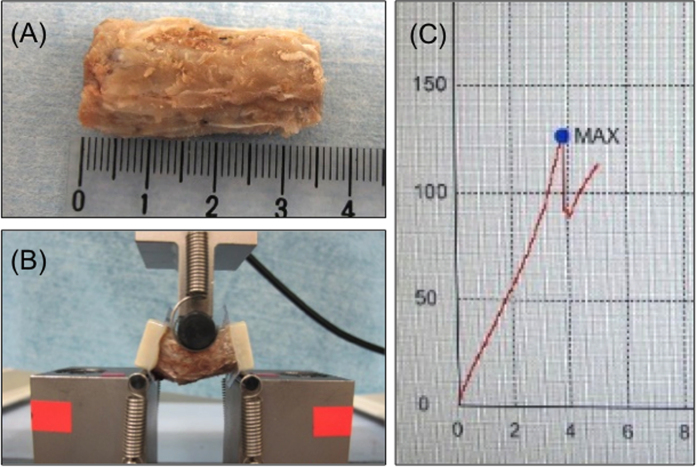
Mechanical strength evaluation: three-point bending test. (**A**) Harvested lumbar spine (L4–L6). (**B**) Three-point bending. (**C**) Representative plotting for initial peak pressure measurement.

L4–L6 lumbar spine specimen (3.5 cm in length) were harvested from 25 rats (five per group) (Fig. 3A), and both sides of the specimen were fixed with a plastic holder (Cryomold #2, Sakura Finetek, Tokyo, Japan) and set on a three-point bending test device (Shimadzu, Tokyo, Japan), as is shown in Fig. 3B, at room temperature within the day of sacrifice to avoid possible temporal alteration in rigidity.

Continuous pressure was applied on the specimen under real-time monitoring. No rotation or slipping of the specimen has been confirmed during the experiment. The bones were preloaded to 10 N at a speed of 0.1 mm/s and allowed to adapt for 10 s^22^. The fracture sites were confirmed after mechanical strength measurement (Fig. 3C) and hematoxylin and eosin staining of the specimen.

#### Statistical Analysis

The number of rats with bone union was presented with 95% confidence intervals, and the differences between the efficiencies of union in each group at 4 and 8 weeks after surgery were analyzed using Mann–Whitney U test. All other data were expressed as means and standard deviations. The correlation of bone volume and state of bone union was described by the correlation coefficient. The differences in bone volumes and trabecular bones were analyzed using analysis of variance with Bonferroni post hoc correction. The differences in growth factor concentrations were analyzed using unpaired t tests. The criterion for significance was p < 0.05.

## Results

### Confirmation of PRP

#### Platelet counts

The mean platelet count was 96.5 ± 8.6/μL in blood and 482.6 ± 24.8/μL in PRP (Table 1). The platelet count in fresh PRP was about 4.9 times greater and was significantly higher than that in blood (p < 0.05).

#### Concentration of growth factors

The mean concentration of PDGF-BB was 1.6 ± 0.4 ng/mL in blood and 23.1 ± 7.2 ng/mL in PRP. The mean concentration of TGF-β1 was 64.3 ± 23.2 ng/mL in blood and 542.7 ± 461.6 ng/mL in PRP (Table 2). The concentrations of these growth factors were significantly higher in PRP than in blood (p < 0.05), and the growth factor concentration in FD-PRP was almost equal to that in fresh PRP. In FD-PRP, PDGF-BB was 19.2 ± 12.4 ng/mL and TGF-β1 was 490.1 ± 332.6 ng/mL.

#### Evaluation of bone union

**Figure 4:**
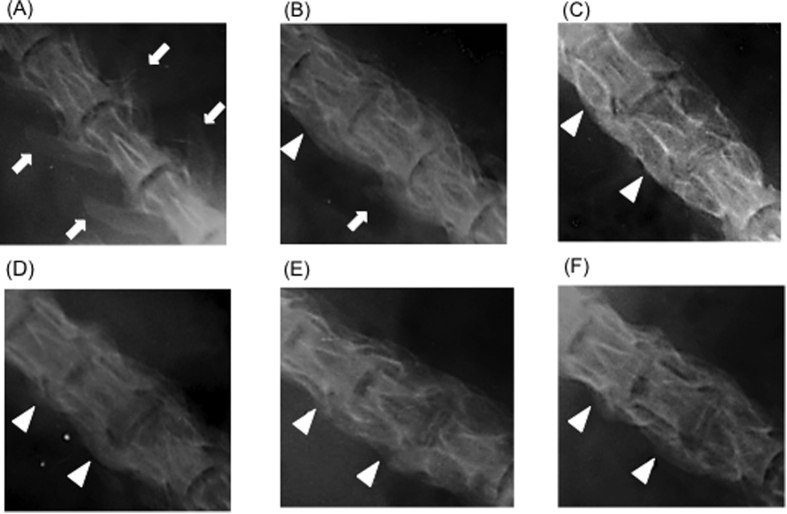
Anteroposterior radiographs of the spinal segment of the platelet-rich plasma group 4 weeks after the surgery. (**A**) Sham group. (**B**) Artificial bone–alone group. (**C**) Autologous bone group. (**D**) Artificial bone + fresh-PRP group. (**E**) Artificial bone + FD-PRP group. (**F**) Artificial bone + BMP group. Arrowheads indicate the part of bone union, and arrows show the transverse process shadow. The FD-PRP group showed greater bone formation compared with the other groups.

**Figure 5:**
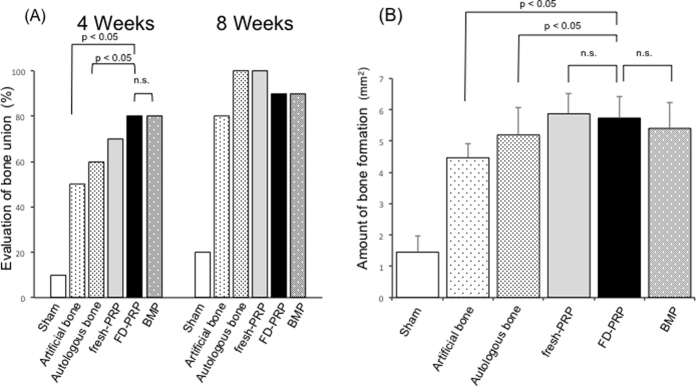
(**A**) Evaluation of bone union. The FD-PRP and BMP groups showed earlier bone formation compared with the artificial bone–alone and autologous bone groups. (**B**) Amount of bone formation 8 weeks after the surgery. n.s., no significance. The FD-PRP group showed more bone formation (p < 0.05), comparable to the fresh-PRP and BMP groups.

The transverse process contours in the sham group 4 weeks after the surgery provided the control image (Fig. 4A). In artificial bone–alone rats (Fig. 4B), mottled contours were observed between the transverse processes and complete continuity was noted in 5 (50%) of 10 animals. In the autologous bone group (Fig. 4C), bone union was observed in 5 (50%) of 10 animals. In the FD-PRP and BMP groups (Fig. 4E,F), 8 (80%) of 10 rats showed confirmed bone union. The transverse process shadow disappeared and formed a lump, and the FD-PRP and BMP groups showed greater bone formation compared with the autologous bone group. At 8 weeks, more rats in the five groups showed bone union than the rats in the sham group (Fig. 5A).

A greater percentage of rats in the FD-PRP and BMP groups showed bone union at 4 weeks compared to the other groups.

#### Histological Analysis

##### Bone formation

At 8 weeks postoperatively, the region of bone graft showed a lump, including the region of the transverse processes, vertebral arch, and spinous process. The bone graft was subsequently replaced by new bone tissue, and remodeling was observed. The amounts of bone formation were as follows (in mm^2^): sham group, 1.45 ± 0.51; artificial bone–alone group, 4.47 ± 0.47; autologous bone group, 5.19 ± 0.89; fresh-PRP group, 5.87 ± 0.64; FD-PRP group, 5.73 ± 0.68; BMP group, 5.4 ± 0.82 (Fig. 5B). The fresh-PRP group showed maximum bone formation, followed by the FD-PRP group.

##### Trabecular bone

**Figure 6:**
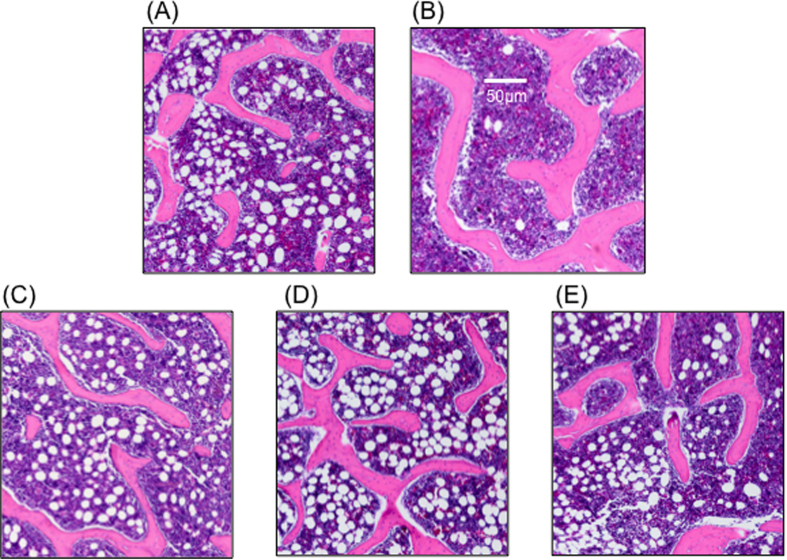
Histological images of trabecular bone. (**A**) Artificial bone–alone group. (**B**) Autologous bone group. (**C**) Artificial bone + fresh-PRP group. (**D**) Artificial bone + FD-PRP group. (**E**) Artificial bone + BMP group. Trabecular bone formation of the FD-PRP group consisted of a tangled structure with more thin branches, compared with the autologous bone group. The trabecular bone formation is similar to that of the fresh-PRP and BMP groups.

Characteristic histological findings in the trabecular bone were observed in each group (Fig. 6). The artificial bone–alone group showed a small plaque. The autologous bone group showed more thickness and lesser branching. The fresh-PRP group, FD-PRP group and the BMP group showed thinning and branching.

##### Total area ratio

**Figure 7:**
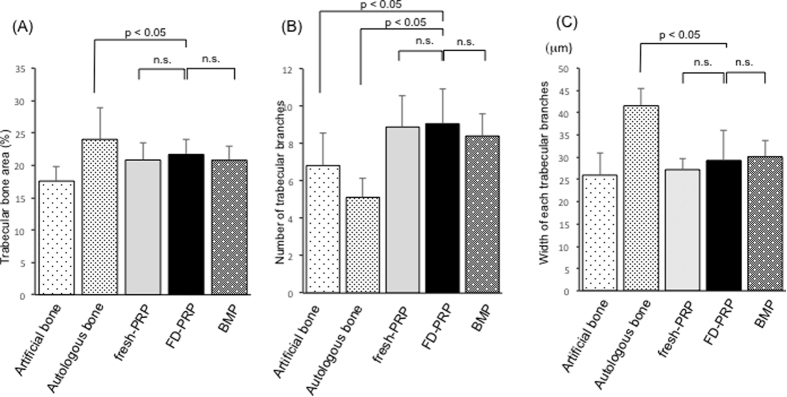
(**A**) Total area ratio: the FD-PRP group was slightly inferior to the autologous bone group. (**B**) Number of trabecular branches: the FD-PRP group had almost the same results as the fresh-PRP and BMP groups but had more branches compared with the artificial bone–alone and autologous bone groups. (**C**) Quantified value of the trabecular width. The trabecular branches in the FD-PRP group formed significantly thinner branches compared with those in autologous bone groups.

The percentage of the trabecular bone area (Fig. 7A) in the each field of view was 17.6 ± 2.3% in the artificial bone–alone group, 24 ± 4.9% in the autologous bone alone group, 20.8 ± 2.6% in the fresh-PRP group, 21.7 ± 2.4% in the FD-PRP group, and 20.8 ± 2.2% in BMP group. The FD-PRP group was inferior to that of the autologous bone group in terms of percentage of trabecular bone area.

##### Number of trabecular branches

The number of trabecular branches (Fig. 7B) of the remodeling part was 6.8 ± 1.7 in the artificial bone–alone group, 5.1 ± 1.0 in the autologous bone group, 8.9 ± 1.7 in the fresh-PRP group, 9.1 ± 1.8 in the FD-PRP group, and 8.4 ± 1.2 in the BMP group. The FD-PRP group showed almost the same results as the fresh-PRP and BMP groups, and more branches compared with the artificial bone–alone and autologous bone groups.

##### Width of each trabecular branches

The width of each trabecular branches (Fig. 7C) in the remodeling bone was 25.9 ± 4.9 μm in the artificial bone–alone group, 41.5 ± 3.9 μm in the autologous bone group, 27.2 ± 2.5 μm in the fresh-PRP group, 29.3 ± 6.7 μm in the FD-PRP group, and 30.1 ± 3.7 μm in the BMP group.

Trabecular branches in the FD-PRP group formed significantly formed thinner branches compared with those in autologous bone groups.

##### Mechanical Strength Examination

**Figure 8:**
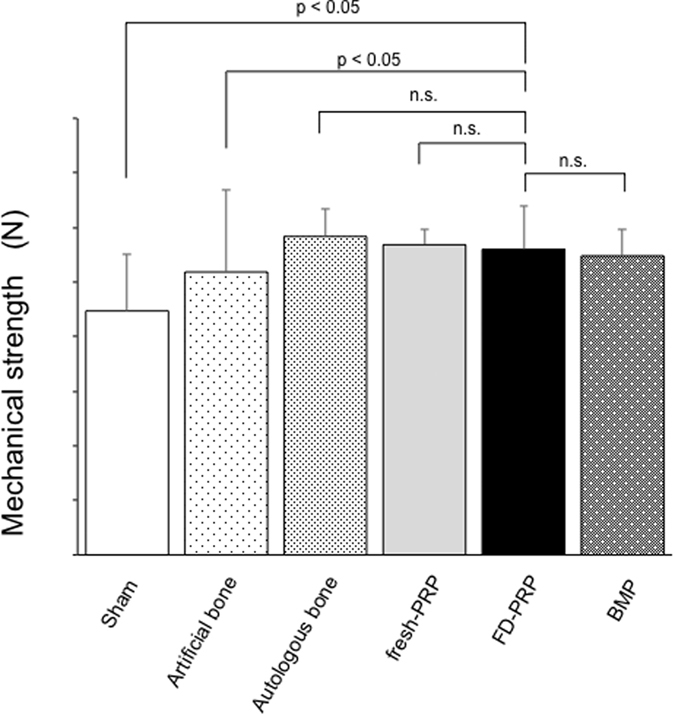
Mechanical strength evaluation using three-point bending test. n.s. no significance. The FD-PRP group was significantly stronger than the sham group and the artificial bone–alone group (p < 0.05), but comparison with the autologous bone group showed no significance.

Figure 8 demonstrates the result of strength measurement. The average values of the initial peak pressure were as follows: sham group, 89.4 ± 20.7N; artificial bone–alone group, 103.7 ± 30.1N; autologous bone group, 116.7 ± 10N; fresh-PRP group, 113.7 ± 5.8N; FD-PRP group, 112.0 ± 15.6N; BMP group, 109.6 ± 9.7N. The FD-PRP group was significantly stronger than that of the artificial bone–alone group (p < 0.05) and slightly inferior compared with the autologous bone group with no significance. The fracture locations in the spinal tissue specimen in all cases were observed to be on the endplate of the vertebral body cartilage.

##### Complications

No significant complications such as infection and tumor generation/formation were observed in the FD-PRP group. An accumulation of inflammatory cells was seen at the bone remodeling site in two rats in the BMP group.

## Discussion

The current study showed that application of FD-PRP combined with artificial bone significantly accelerated bone fusion comparable to that of fresh-PRP and BMP. In addition, trabecular bone formation showed a more tangled structure with many thin branches at the stage of 8 weeks after the surgery with comparable rigidity to autologous bone.

The resulting bone union in this study was considered to have been achieved with PRP, which has been reported to contain a large number of growth factors and to exert tissue repair effects^23^,^24^,^25^. In the current study, FD-PRP prepared 8 week before the surgery accelerated bone formation with artificial bone in a rat PLF model.

The details of the properties of the trabecular bone achieved by the application of FD-PRP were considered to be significant. Histologically, in all of the groups except the sham, bone grafts showed remodeling and fusion with new bone structures. In evaluating the trabecular bone strength, the thickness, number of branches, and total area are important factors^26^, although no previous reports have compared the detailed properties on trabecular bone. The FD-PRP group showed characteristic findings of large stitch structures with thin branches, whereas the total area is slightly inferior to that of the autologous bone group. However, the results of the mechanical strength test of FD-PRP–treated spine showed comparable strength to autologous bone. This result indicates that FD-PRP achieved more rigidity by more tangled trabecular structures rather than thick ones as well as accelerated bone union. Furthermore the current study adopted BMP as the positive control, as its bone formation effect has already been confirmed and it is being commonly used clinically; however, it is also known for its risks such as tumor formation and excessive inflammation and it is expensive as well^27^,^28^,^29^, leading to its unavailability in some countries. The result of the current study that the FD-PRP group showed comparable to BMP in terms of bone union ability can be extremely important, especially in those countries where BMP is unavailable and even in countries where BMP is available, considering its possible risk. Furthermore, PRP is safer because it is made from the patient’s own blood. Clinical usage of PRP has been reported to have few significant complications^7^, which coincides with the finding of the current study. The fact that FD-PRP achieved the similar tendency for trabecular formation and mechanical strength to BMP indicates that FD-PRP achieves accelerated bone formation in the osteoinductive manner as BMP does^30^. This should be investigated in detail in a future study.

The current study suggests the usefulness of FD-PRP, as it can be prepared and stored beforehand, unlike fresh PRP, which should be prepared immediately just before the time of an operation because of the short half-life of its growth factors. In the future, clinical trials on the safety and efficacy of FD-PRP on spine surgery should be performed, considering possible application to other fields of orthopedic surgery such as trauma.

The current study includes some limitations. First, we should be more specific in evaluating the histology. For instance, with respect to bone union between the transverse processes, only simple radiography was performed. We should be more specific in evaluating the bone union using other radiographic modalities such as micro-computed tomography scan in a future study. The evaluation of the amount of bone formation has a slight inaccuracy because parts of the original spinous process and lamina were included. Second, we should explore the result at earlier or later time points other than 4 and 8 weeks. Third, a single-animal model using rat can be inadequate in proving the efficacy of FD-PRP. Considering future clinical applications, we should examine other species such as rabbits and dogs, which are used in traditional bone fracture experiments and have longer bone-union period similar to humans. Fourth, with respect to bone strength, only the three-point bending test was performed. We should be more specific in evaluating bone strength using other tests, such as torsion test, in a future study^21^.

Furthermore, we evaluated the effectiveness of FD-PRP, focusing its ability for bone union and formation, and it should be evaluated in detail from the bone metabolic point of view, including the reaction of osteoclasts and osteoblasts. We also mainly focused on the effectiveness of FD-PRP focusing on the ability for bone union and formation in the current study. In terms of bone formation, new bone formation should also be evaluated. These should be investigated for more robust evidence.

In conclusion, the current study indicated that FD-PRP enabled earlier bone union in the rat PLF model. The amount of bone formation and trabecular bone remodeling was increased in the FD-PRP–treated samples, with confirmed more trabecular branches and biomechanical rigidity. Mechanical strength was stronger when FD-PRP was added to artificial bone at 8 weeks after surgery, with bone union and rigidity comparable to fresh-PRP and BMP.

## Additional Information

**How to cite this article**: Shiga, Y. *et al*. Freeze-Dried Platelet-Rich Plasma Accelerates Bone Union with Adequate Rigidity in Posterolateral Lumbar Fusion Surgery Model in Rats. *Sci. Rep.* **6**, 36715; doi: 10.1038/srep36715 (2016).

**Publisher’s note:** Springer Nature remains neutral with regard to jurisdictional claims in published maps and institutional affiliations.

## Figures and Tables

**Table 1:** Mean platelet counts (/μL).

Blood	PRP
96.5 ± 8.6	482.6 ± 24.8

Values are presented as mean ± standard deviation. PRP, platelet-rich plasma.

**Table 2:** Concentration of growth factors (ng/mL).

	Blood	PRP	FD-PRP
PDGF-BB	1.6 ± 0.4	23.1 ± 7.2	19.2 ± 12.4
TGF-β1	64.3 ± 23.2	542.7 ± 461.6	490.1 ± 332.6

Values are presented as mean ± standard deviation.

PRP, platelet-rich plasma; FD-PRP, freeze-dried platelet-rich plasma; PDGF-BB, platelet-derived growth factor BB; TGF-β1, transforming growth factor β1.
